# Persistent Associations between Maternal Prenatal Exposure to Phthalates on Child IQ at Age 7 Years

**DOI:** 10.1371/journal.pone.0114003

**Published:** 2014-12-10

**Authors:** Pam Factor-Litvak, Beverly Insel, Antonia M. Calafat, Xinhua Liu, Frederica Perera, Virginia A. Rauh, Robin M. Whyatt

**Affiliations:** 1 Department of Epidemiology, Mailman School of Public Health, Columbia University, New York, New York, United States of America; 2 National Center for Environmental Health, Centers for Disease Control and Prevention, Atlanta, Georgia, United States of America; 3 Department of Biostatistics, Mailman School of Public Health, Columbia University, New York, New York, United States of America; 4 Department of Environmental Health Sciences, Columbia Center for Children’s Environmental Health, Mailman School of Public Health, Columbia University, New York, New York, United States of America; 5 Heilbrunn Department of Population and Family Health, Mailman School of Public Health, Columbia University, New York, New York, United States of America; Institute for Health & the Environment, United States of America

## Abstract

**Background:**

Prior research reports inverse associations between maternal prenatal urinary phthalate metabolite concentrations and mental and motor development in preschoolers. No study evaluated whether these associations persist into school age.

**Methods:**

In a follow up of 328 inner-city mothers and their children, we measured prenatal urinary metabolites of di-n-butyl phthalate (DnBP), butylbenzyl phthalate (BBzP), di-isobutyl phthalate (DiBP), di-2-ethylhexyl phthalate and diethyl phthalate in late pregnancy. The Wechsler Intelligence Scale for Children, 4th edition was administered at child age 7 years and evaluates four areas of cognitive function associated with overall intelligence quotient (IQ).

**Results:**

Child full-scale IQ was inversely associated with prenatal urinary metabolite concentrations of DnBP and DiBP: b = −2.69 (95% confidence interval [CI] = −4.33, −1.05) and b = −2.69 (95% CI = −4.22, −1.16) per log unit increase. Among children of mothers with the highest versus lowest quartile DnBP and DiBP metabolite concentrations, IQ was 6.7 (95% CI = 1.9, 11.4) and 7.6 (95% CI = 3.2, 12.1) points lower, respectively. Associations were unchanged after control for cognition at age 3 years. Significant inverse associations were also seen between maternal prenatal metabolite concentrations of DnBP and DiBP and child processing speed, perceptual reasoning and working memory; DiBP and child verbal comprehension; and BBzP and child perceptual reasoning.

**Conclusion:**

Maternal prenatal urinary metabolite concentrations measured in late pregnancy of DnBP and DiBP are associated with deficits in children’s intellectual development at age 7 years. Because phthalate exposures are ubiquitous and concentrations seen here within the range previously observed among general populations, results are of public health significance.

## Introduction

Phthalates are a class of high production chemicals widely used as plasticizers and additives in consumer and personal care products [1]. Many phthalates are endocrine disruptors which may operate through multiple mechanisms including perturbations in thyroid hormone and testosterone levels [2], [3]. Exposures to phthalates are ubiquitous [4], [5]. Urinary concentrations of phthalate metabolites are used as internal dosimeters because urinary enzymatic activity is negligible [6]; thus metabolite concentrations in urine reflect an individual’s internal exposure to phthalates, rather than phthalate contaminants introduced during sample collection and processing. Prior studies have shown moderate reproducibility (i.e. intraclass correlation coefficients ranging from approximately 0.20 to 0.77) for measurements of several phthalate metabolite concentrations in repeat spot urine samples [5], [7]–[9].

Limited epidemiologic studies have reported inverse associations between phthalate metabolites in maternal prenatal urine and child mental, motor and behavioral development [5], [10]. Previously, we reported that maternal prenatal urinary concentrations of mono-n-butyl phthalate (MnBP) and monoisobutyl phthalate (MiBP), the main metabolites of di-n-butyl phthalate (DnBP) and di-isobutyl phthalate (DiBP), respectively, were inversely associated with child age 3 year motor development and increased the risk of motor delay [5]. Among girls, MiBP was also inversely associated with mental development [5]. Experimental animal studies find inverse associations between prenatal exposure to di-2-ethylhexyl phthalate (DEHP) and DnBP and learning and memory in the offspring [11], [12]. No prior studies have evaluated associations between prenatal phthalate exposures on child intelligence quotient (IQ) in school-age children. However, a cross-sectional study of Korean 3^rd^ and 4^th^ grade children found inverse associations between DEHP metabolites in child urine and IQ [13]. Based on these findings, we hypothesized that prenatal phthalates exposures would be inversely associated with child IQ at age 7 years.

## Methods

We studied 328 inner-city women and their 7-year old children from the Columbia Center for Children’s Environmental Health (CCCEH) longitudinal birth cohort of 727 pregnant women who delivered between 1998 and 2006. The original aim of the cohort was to examine the associations between exposure to air pollutants and pregnancy outcomes and child development. Enrollment, exclusion criteria, and a description of the cohort have been described previously [14]. Women 18–35 years old who self-identified as either African American or Dominican were enrolled through prenatal clinics associated with Harlem and New York Presbyterian Hospitals. Women were excluded if they reported active smoking, use of other tobacco products or illicit drugs, had diabetes, hypertension or known HIV, had their first prenatal visit after the 20th week of gestation or had resided in the study area for less than one year prior to pregnancy. Mother-child pairs were selected for participation in the current study if phthalate metabolite concentrations had been measured in spot urine samples collected during pregnancy and if the child had completed the Wechsler Intelligence Scale for Children, 4th edition (WISC-IV) at age 7 years. We excluded women with active smoking during pregnancy verified by maternal and/or umbilical cord plasma cotinine >15 ng/ml at delivery (n = 30), no or insufficient urine for measurement of phthalate metabolites (n = 286), and those lost to follow-up prior to child age 7 years (n = 83). Among women who had prenatal phthalate measurements, the retention rate was 80% at the 7-year follow-up. The 328 study subjects did not differ significantly from the remaining subjects in the CCCEH cohort in terms of demographics (race/ethnicity, maternal marital status, education level, household income, proportion receiving Medicaid), prenatal alcohol consumption, child sex, gestational age, and birth weight (all *p*-values>0.05). Moreover, study children did not differ from the remaining children with respect to mental and motor development scores at age 3 years.

### Ethics Statement

Institutional review boards at the Columbia University Medical Center and the Centers for Disease Control and Prevention (CDC) approved the study and all consent procedures for the study. Written informed consent was obtained from all participating mothers, who also provided written informed consent on behalf of their children, and written informed assent was obtained from all children starting at age 7 years.

### Urine sample collection and phthalate measurements

Spot urine samples were collected during the 3^rd^ trimester of pregnancy (average 34.0±3.0 weeks, median 33.9) and from the children at ages 3 (n = 241) and 5 (n = 277) years**.** Samples were analyzed for metabolites of 5 phthalates (DnBP, BBzP, DiBP, DEHP and diethylphthalate) at the CDC as described [15]. Specific gravity was measured in the urine samples using a handheld refractometer and used to control for urinary dilution (Atago PAL 10-S, Bellevue, WA) [7].

As a measure of reliability, we calculated intraclass correlation coefficients (ICCs) for the phthalate metabolites in serial spot urine samples collected biweekly from 48 women in the CCCEH cohort over 6–8 weeks late in pregnancy (*n* = 135 samples, 2–4 repeats per woman). Adjusting for specific gravity, ICCs were 0.77 for MBzP, 0.65 for mono-*n*-butyl phthalate (MnBP), and 0.60 for monoisobutyl phthalate (MiBP) and ranged from 0.27 to 0.42 for the DEHP metabolites [5].

### Measures of child mental development

The Wechsler Intelligence Scale for Children, 4th edition (WISC-IV) [16] was administered to children at age 7 years. The instrument measures four areas of mental functioning that are associated with, but distinct from, overall IQ. The Verbal Comprehension Index is a measure of verbal concept formation; the Perceptual Reasoning Index measures nonverbal and fluid reasoning; the Working Memory Index assesses children’s ability to memorize new information, hold it in short-term memory, concentrate, and manipulate information; and the Processing Speed Index assesses ability to focus attention and quickly scan, discriminate, and sequentially order visual information. Full-Scale IQ score combines the four composite indices. All WISC-IV scales are standardized to a mean of 100 and standard deviation (SD) of 15. The WISC-IV has been shown in prior research to be sensitive to effects of low-dose neurotoxicant exposures on cognition [17]–[19].

### Model covariates

Information on potential confounders was gathered by questionnaires administered to the mother during pregnancy and at various postnatal intervals by trained bi-lingual interviewers and by review of maternal and infant medical records. Variables of interest included race/ethnicity, maternal education and marital status, household income, parity, gestational age, birth weight, child sex, breastfeeding history, exposure to tobacco smoke in the home, prenatal alcohol consumption, and prenatal psychosocial factors including maternal self-report of hardship during pregnancy (i.e., lack of food, clothing, housing, gas or electricity, or medicines) and satisfaction with overall living conditions. Maternal demoralization was measured by the 27-item Psychiatric Epidemiology Research Instrument-Demoralization Scale [20]. Maternal intelligence was assessed by the Test of Non-Verbal Intelligence, third edition [21], a language-free measure of general intelligence, which is relatively stable and free of cultural bias. The quality of the care-taking environment was measured by the Home Observation for Measurement of the Environment (HOME) scale [22] at child age 38.4±6.2 months.

### Statistical analysis

Linear regression models were used to examine relationships between prenatal exposures to the five phthalates (assessed from the urinary metabolite concentrations) and WISC-IV outcomes. Phthalate metabolite concentrations below the limit of detection (LOD) (one for monobenzyl phthalate (MBzP), one for mono-isobutyl phthalate (MiBP) and 53 for mono-2-ethylhexyl phthalate (MEHP)) were assigned a value of LOD/√2 [23]. The distributions of the phthalate metabolite concentrations were right skewed and transformed using the natural logarithm to improve model fitting and reduce the influence of extreme values. In our analyses, each metabolite was considered as a continuous variable and was categorized into quartiles to explore the shape of the dose response relationship. The final regression models included covariates that were *a priori* potential confounders based on previous literature and a directed acyclic graph, and that were associated with at least one WISC-IV subscale or the total WISC-IV score [5], [17], [18]. Missing values for covariates were imputed as follows: *a*) twelve missing values for maternal IQ were imputed by a linear regression model with race/ethnicity, maternal education and age as predictors (model *R* ^2^ = 0.13); *b*) twenty missing observations for the HOME scale were imputed by linear regression model with race/ethnicity, maternal education and IQ, and household income as predictors (model *R* ^2^ = 0.18); *c*) ten missing observations for prenatal maternal alcohol consumption were given a category of missing. Sensitivity analyses were conducted for observations with no missing data (reducing the sample size to 290); results were essentially unchanged from those reported here. We used maternal urinary concentrations of mono-2-ethyl-5-hydroxyhexyl phthalate (MEHHP) as the proxy for exposure to DEHP. All DEHP metabolites were highly correlated with each other (Spearman correlation coefficient r≥0.8) [25]. In a sensitivity analysis results using MEHP as the DEHP exposure proxy were essentially the same. Analyses were repeated using the molar sum of all four DEHP metabolites, also with essentially the same results. Urinary specific gravity was included in all models to control for dilution ^7^. We evaluated confounding in two ways. First, we constructed a directed acyclic graph to determine potential confounders. Second, for each potential confounder, we assessed the change in the estimated regression coefficient between the exposure of interest (i.e. specific phthalate metabolite) and outcome (i.e. either the WISC IQ measure or the WISC subscale measure) with and without the potential confounder. We included variables in the model if their inclusion changed the estimated regression coefficient between exposure and outcome by more than.5 standard deviation units. To evaluate whether sex of the child was an effect modifier, we conducted analyses separately by sex, and assessed whether the estimated coefficients differed using the Wald test. In secondary analyses, we included a measure of cognitive performance at age 3 years (the Mental Development Index (MDI) from the Bayley Scales of Infant Development-second edition (1993). Additionally, we evaluated whether child age 3 and 5 years urinary phthalate metabolite concentrations were associated with WISC-IV outcomes at age 7 years. We also evaluated whether other contaminants, namely lead, chlorpyrifos, and polycyclic aromatic hydrocarbons (PAHs), were potential confounders. With the exception of a negative correlation between PAHs and one phthalate metabolite (MiBP) (r = −0.13, p = .02), no correlations were found between any other contaminant and phthalate metabolite. In a sensitivity analysis, we included PAHs in the regression model relating MiBP to the cognitive outcomes; the magnitudes of the regression coefficients became stronger and more statistically significant. Analyses were conducted using SAS (version 9.3 SAS Institute Inc., Cary, NC).

## Results

Maternal sociodemographic characteristics, infant birth and child characteristics and distributions of model covariates and outcome variables are presented in Table 1. Eighteen children (5.5%) were administered the test in Spanish. Table 2 shows the distribution of the urinary phthalate metabolite concentrations in maternal prenatal spot samples. Metabolites were detected in 99.7–100% of the samples, except for MEHP in which 16% of the measures were below the LOD. Nevertheless, for the 53 measures that were below the LOD, using the actual concentrations or the assignment of a value (1/√2)×LOD produced essentially the same results. Spearman correlation coefficients between the specific gravity adjusted maternal prenatal metabolite concentrations ranged from 0.15 (for monoethyl phthalate (MEP) and both MBzP and MEHHP) to 0.63 (for MiBP and MnBP). Correlations for the urinary phthalate metabolite concentrations in child age 3 and 5 year samples were similar (data not shown). However, correlations between each phthalate metabolite across ages (prenatal, age 3 and 5 years) were not statistically significant. The correlation between Bayley MDI score and full scale IQ at age 7 was 0.43 (p<.0001) and between maternal IQ and full scale IQ at age 7 was 0.26 (p<.0001).

**Table 1:** Subject demographics, distribution of model covariates, and outcome variables (N = 328).

Characteristic	Value (%)
Maternal age at prenatal interview (yr)	25.3±4.8
Ethnicity	
African American	215 (34.5)
Dominican or other Hispanic	113 (65.5)
Maternal education	
<High school degree	119 (36.3)
≥High school diploma or general educational development (GED)	209 (63.7)
Marital status	
Never married	220 (67.1)
Ever Married^a^	108 (32.9)
Maternal IQ (n = 316)	84.6±13.3
HOME scale (n = 308)	39.2±6.3
Prenatal alcohol consumption (N = 318)	82 (25.8)
Child sex	
Male	155 (47.3)
Female	173 (52.7)
Child age at WISC-IV (yr)	7.05±0.20
**WISC-IV Outcome variables**	
Full Scale Composite Score	97.1±13.1
Perceptual Reasoning Composite Score	99.3±14.0
Processing Speed Composite Score	98.9±14.6
Verbal Comprehension Composite Score	94.4±12.7
Working Memory Composite Score	98.5±14.9

Values are mean ± SD or n (percent). Unless indicated, N = 328.

a Includes living with same partner for >7 years.

**Table 2:** Distribution of Phthalate metabolites (ng/ml) in maternal spot urine during the third trimester of pregnancy (n = 328).

Metabolite	Mean	95% CI	LOD*	%<LOD	Range	25%	Median	75%
MnBP	37.6	(33.5, 42.3)	0.6	0	1.2–1,110	19.4	38.0	79.8
MBzP	13.4	(11.6, 15.4)	0.22	0.3	ND–550.4	5.8	14.4	30.0
MEHHP	22.3	(19.4, 25.5)	0.7	0	1.1–1750	10.6	21.8	47.2
MEHP	4.95	(4.2, 5.7)	1.2	16.2	ND–613	1.9	4.9	12.4
MEP	160.5	(140.4, 183.4)	0.53	0	7.8–6045.6	69.9	141.5	334.1
MiBP	9.1	(8.1. 10.2)	0.3	0.3	ND–374.4	5.0	9.2	19.0

ND = not detected.

In the total cohort (table 3), full scale IQ was inversely associated with log*_e_*MnBP (b = –2.69 [95% CI = –4.33, –1.05]) and log*_e_*MiBP (b = –2.69 [95% CI = –4.22, –1.16]) but not with the other phthalate metabolites. Additonally, log*_e_*MnBP, log_e_MiBP and log*_e_*MBzP were significantly inversely associated with perceptual reasoning, log*_e_*MnBP and log_e_MiBP with processing speed, log_e_MiBP with verbal comprehension, and log*_e_*MnBP and log_e_MiBP with working memory. There were no significant associations between maternal MEHP, MEHHP or MEP concentrations and any of the WISC-IV scales. There were some differences in the estimated regression coefficients relating the exposures to outcomes between boys and girls, although with one exception none reached statistical significance. Specifically, associations between MnBP and full scale IQ and perceptual reasoning appeared stronger among girls than boys, and associations between MnBP and processing speed appeared stronger among boys. Additionally, associations between MBzP and perceptual reasoning and between MiBP and verbal comprehension appeared stronger among boys. MnBP was associated with working memory and the size of the associations was significantly larger among girls than boys (p = 0.02 Wald test). Controlling for postnatal year three and year five phthalate metabolites concentrations did not alter the association between prenatal phthalate exposures and the WISC outcomes at age 7 years.

**Table 3:** Estimated adjusted regression coefficients relating maternal prenatal urinary phthalate concentrations to the WISC-IV full scale IQ and subscales at child age 7 years.

	Β-Coefficient (95% CI)								Child sex
	Total		Girls				Boys		Difference
Metabolite (log base e)	(n = 328)		(n = 173)				(n = 155)		*p*-value^@^
**Full Scale**									
MBnP	−2.69	(−4.33,−1.05)**	−3.15		(−5.44,−0.87)**	−1.89		(−4.34, 0.56)	0.46
MBzP	−1.18	(−2.40, 0.05)	−0.84		(−2.52, 0.83)	−1.48		(−3.35, 0.38)	0.61
MEHHP	0.16	(−1.16, 1.48)	0.34		(−1.35, 2.03)	0.26		(−1.87, 2.40)	0.96
MEHP	−0.30	(−1.42, 0.82)	−0.05		(−1.53, 1.42)	0.09		(−1.92, 2.11)	0.74
MEP	−0.17	(−1.46, 1.13)	−0.75		(−2.53, 1.03)	0.40		(−1.53, 2.33)	0.39
MiBP	−2.69	(−4.22,−1.16)**	−2.38		(−4.50,−0.26)*	−2.92		(−5.17,−0.67)*	0.73
**Perceptual Reasoning**									
MnBP	−2.58	(−4.40,−0.76)**	−3.55		(−5.96,−1.14)**	−1.50		(−4.36, 1.35)	0.28
MBzP	−1.65	(−3.00,−0.30)*	−1.13		(−2.89, 0.64)	−2.45		(−4.60,−0.31)*	0.35
MEHHP	0.18	(−1.28, 1.64)	0.03		(−1.76, 1.82)	0.56		(−1.92, 3.04)	0.73
MEHP	−0.07	(−1.30, 1.17)	−0.09		(−1.65, 1.48)	0.01		(−2.00, 2.03)	0.94
MEP	−0.62	(−2.05, 0.81)	−0.67		(−2.56, 1.22)	−0.57		(−2.81, 1.67)	0.95
MiBP	−2.41	(−4.11,−0.71)*	−2.39		(−4.64,−0.14)*	−2.41		(−5.05, 0.23)	0.99
**Processing Speed**									
MnBP	−2.01	(−3.91,−0.11)*	−1.29		(−4.04, 1.45)	−2.85		(−5.63,−0.08)*	0.43
MBzP	−0.47	(−1.88, 0.95)	−0.74		(−2.72, 1.23)	−0.02		(−2.17, 2.12)	0.63
MEHHP	−0.41	(−1.91, 1.10)	−0.84		(−2.83, 1.15)	0.25		(−2.19, 2.68)	0.50
MEHP	−0.95	(−2.23, 0.33)	−0.93		(−2.67, 0.80)	−0.94		(−2.92, 1.03)	0.99
MEP	0.54	(−0.95, 2.03)	−0.26		(−2.37, 1.84)	1.21		(−0.99, 3.41)	0.34
MiBP	−1.94	(−3.72,−0.17)*	−1.94		(−4.46, 0.58)	−2.10		(−4.70, 0.50)	0.93
**Verbal Comprehensionn** **oCnsion**									
MnBP	−1.52	(−3.06, 0.02)	−1.06		(−3.29, 1.16)	−1.64		(−3.90, 0.62)	0.72
MBzP	−0.78	(−1.92, 0.36)	−0.46		(−2.06, 1.14)	−1.07		(−2.79, 0.66)	0.61
MEHHP	0.46	(−0.76, 1.69)	0.86		(−0.75, 2.47)	0.09		(−1.88, 2.06)	0.55
MEHP	−0.28	(−1.32, 0.76)	−0.01		(−1.42, 1.40)	−0.60		(−2.20, 1.00)	0.58
MEP	0.10	(−1.30, 1.11)	−0.71		(−2.42, 0.99)	0.52		(−1.26, 2.31)	0.32
MiBP	−2.08	(−3.51,−0.65)**	−1.05		(−3.10, 1.00)	−3.04		(−5.11,−0.98)**	0.18
**Working Memory**									
MnBP	−2.57	(−4.55,−0.59)**	−4.73		(−7.53,−1.93)**	−0.07		(−2.92, 2.78)	0.02
MBzP	−0.68	(−2.16, 0.80)	−0.59		(−2.67, 1.48)	−0.50		(−2.68, 1.67)	0.95
MEHHP	0.36	(−1.23, 1.94)	1.16		(−0.92, 3.25)	−0.29		(−2.76, 2.18)	0.37
MEHP	0.55	(−0.79, 1.89)	1.22		(−0.60, 3.04)	0.09		(−1.92, 2.11)	0.41
MEP	−0.39	(−1.95, 1.17)	−1.06		(−3.26, 1.15)	0.28		(−1.96, 2.52)	0.40
MiBP	−1.98	(−3.84,−0.12)*	−2.53		(−5.16, 0.11)	−1.27		(−3.92, 1.38)	0.51

*p<0.05, **p≤0.01. ^@^Wald Test.

Adjusted model for specific gravity, maternal IQ, ethnicity, alcohol use during pregnancy, education, marital status, total home score, and sex of child.

**Figure 1:**
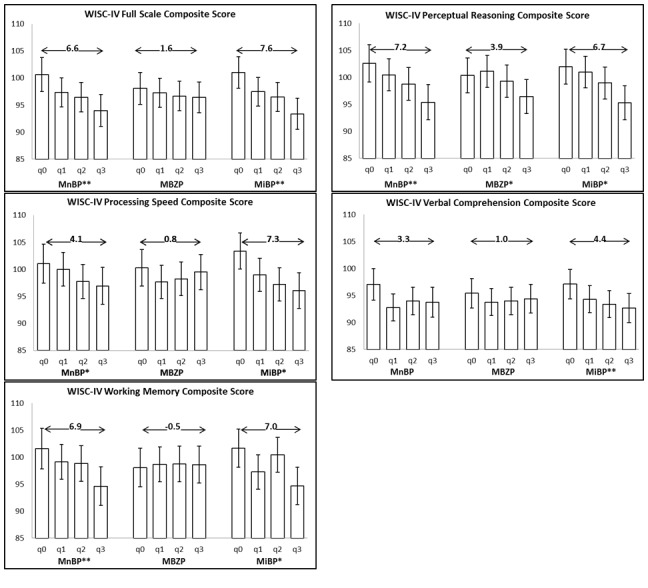
Adjusted mean WISC-IV total score and subtest scored by lowest to highest quartile of maternal prenatal phthalate metabolite concentration (where q0 = lowest quartile, q4 = highest quartile, q2 and q3 intermediate quartiles). Means adjusted for urine specific gravity, maternal IQ, ethnicity, alcohol use during pregnancy, education, marital status, quality of the home environment (HOME score) and sex of child. *p<0.05, **p≤0.01.

Full scale IQ scores among children born to mothers with urinary MnBP and MiBP concentrations in the highest compared to the lowest quartiles were 6.6, 95% CI = (1.89, 11.41) and 7.6, 95% CI = (3.2, 12.1) points lower, respectively (Figure 1). Similar differences were found for the perceptual reasoning, processing speed and working memory scores. Children whose mother had the highest versus lowest concentration of MBzP and MiBP had significantly lower scores on perceptual reasoning (by 3.9 points) and verbal comprehension (by 4.4 points), respectively.

Examination of postnatal phthalate exposure controlling for prenatal exposure suggested specific associations between MBzP measured at age 3 and several of the outcomes. Specifically, we found inverse associations between, log*_e_* MBzP concentrations measured at age 3 were inversely associated with full-scale IQ (b = –1.52; 95% CI = (–2.71, –0.32)), perceptual reasoning (b = –1.68; 95% CI = (–3.01, –0.36)), and working memory (b = –2.2.47; 95% CI = (–3.92, –1.01)) at age 7 years (see Table S1). We observed no associations between concentrations of phthalate metabolites measured at age 5 years and IQ measured at age 7 years; nor did we see any significant associations between the WISC-IV scales and MnBP and MiBP measured at age 3 years. Finally, there was no change in the associations between prenatal urinary phthalate metabolites and any of the WISC outcomes after inclusion of the child age 3 years mental development index (MDI) on the Bayley Scales of Infant Development. Associations also did not change when language of test administration was controlled.

## Discussion

In our follow up study of children prenatally exposed to phthalates, we found significant associations between exposure to DnBP and DiBP and IQ measured at age 7 years, after adjusting for potential confounders. Compared to children born to women in the lowest 25^th^ concentration percentile, children born to women above the 75^th^ concentration percentile for MnBP and MiBP scored 6.6 and 7.6 points lower on 7 year IQ. Similar associations were found between these metabolites and perceptual reasoning, working memory and processing speed subscales of the WISC-IV. Differences in the estimates of association were found in boys and girls. Associations between maternal prenatal MnBP concentrations and child age 7 full scale IQ, perceptual reasoning and working memory were stronger among girls and associations between maternal prenatal MnBP and MiBP concentrations and processing speed and verbal comprehension, respectively, were stronger among boys. We note however, that with one exception the interactions between phthalate metabolites and sex did not reach conventional statistical significance criterions.

These findings extend our earlier observation of associations between prenatal exposure to phthalates and children’s cognitive function and behavior at age 3 years^5^. In the earlier analysis, we found inverse associations between urinary concentrations of MnBP and MiBP and scores on the psychomotor development index (PDI) of the Bayley Scales of Infant Development (BSID) for both boys and girls, and an inverse association between MnBP concentrations and the mental development index (MDI) of the BSID in girls only. Taken together, our findings suggest adverse associations between prenatal phthalate exposure and cognition that persist into the early school years, with potentially meaningful implications for academic performance. We also find associations between MBzP measured at age 3 and full scale IQ, and the perceptual motor and working memory subscales at age 7, suggesting a role of postnatal exposure for specific phthalates.

Several studies have reported associations between prenatal phthalate exposure and neurodevelopment, but the literature is inconsistent regarding the specific phthalate metabolites examined and the finding of sex-specific associations. Engel, et al (2009) [24] found associations in girls, but not boys, between metabolites of high molecular weight phthalates (e.g. DEHP, BBzP) on the Brazelton Neonatal Behavioral Scale administered within five days of delivery. Yolton, et al (2011) [25] reported associations between the urinary concentrations of DEHP metabolites and suboptimal neurological reflexes in boys at 5 weeks of age. Kim, et al (2011) [10] found associations between urinary concentrations of DEHP metabolites and delays in both BSID mental and motor development and urinary concentrations of DnBP metabolite and delays in mental development in 6-month old Korean boys. These three studies are limited by the measure of neurobehavioral assessment, which becomes more reliable as the children age. Tellez-Rojo et al (2013) [26] evaluated prenatal phthalate exposure and repeated BSID scores at ages 2, 2.5 and 3 years in 135 children enrolled in the ELEMENT study in Mexico and found associations with DEHP metabolites in sex-specific analyses only. A final, albeit cross sectional, study found inverse associations between metabolites of DEHP and DnBP and vocabulary development at ages 8–11 years among Korean children [13]. The inconsistent associations regarding the specific phthalates may be due to the variability in age of assessment (the BSID become more stable with increasing age at assessment), the WISC testing different constructs than the BSID, poor adjustment for the correlations between phthalate metabolites, and differences in the concentrations of phthalates in the specific populations. Nevertheless, the consistent pattern of associations between MnBP and MiBP across ages in our cohort lends support to our cognitive findings.

Comparison of the concentrations of phthalate metabolites in our study to those in the last reported NHANES data [27] find, as expected, slightly higher concentrations among women in our sample. However, the confidence intervals in our data and the NHANES data overlap substantially, suggesting that the concentrations in our study are still relevant.

There are several possible mechanisms underlying these associations. Phthalates may act as anti-androgens and lead to disruption in the normal sexual differentiation of the brain [28]–[30]; they may modulate the activity of aromatase in the developing brain and thus interfere with estrogen synthesis [31], [32]; they may interfere with thyroid hormone production [33]–[36], [29], [37]–[41]; and they may disrupt brain dopaminergic activity [42], [43] which is linked to inattention and hyperactivity [44]. These mechanisms may shed light on why the adverse associations are sex specific.

Our study has a number of strengths. First it is a prospective evaluation with assessment of exposure to phthalates not only in the prenatal period, but also at ages 3 and 5 years. It is noteworthy that our associations were primarily limited to prenatal concentrations of phthalate metabolites, with some additional associations seen for age 3 exposures, suggesting that there are critical periods of exposure related to adverse cognitive outcomes. Second, although our sample size was likely not sufficient to estimate sex-specific associations, we did observe several sex specific differences in associations. This is important given that many of the purported mechanisms for these associations are linked to brain concentrations of sex hormones. However, there are also some limitations. We are unable to identify specific times during pregnancy when phthalates could be related to outcomes as urine was only collected from the pregnant women in the third trimester. Because phthalates have a half- life of approximately 12 hours, single, spot urine measures do not reflect long term exposure. We evaluated reproducibility of these urine measures in a sample of 48 women who had repeat urine measures during the third trimester; the ICCs were 0.77 for MBzP, 0.65 for mono-*n*-butyl phthalate (MnBP), and 0.60 for monoisobutyl phthalate (MiBP) and ranged from 0.27 to 0.42 for the DEHP metabolites, indicating moderate reliability over a short time span. Strictly speaking, therefore, our results should be specific to phthalate exposure during the third trimester. Restriction of the study sample to inner-city African American and Hispanics reduces the generalizability of the results, but likely also minimized residual confounding by socioeconomic status and race. We note that we also controlled for a variety of factor known to be associated with child IQ, namely maternal IQ, race/ethnic group, alcohol use during pregnancy, maternal education, marital status, other contaminants and HOME score. Birth weight, another predictor of child IQ, did not change the estimated associations between any phthalate metabolite and IQ. Further work in other ethnic and socioeconomic populations would be needed to generalize these results. We also measured a limited number of phthalate metabolites and thus cannot infer our results to other phthalates. We also could not evaluate the associations between phthalate exposure and school performance as these data are not available. Finally, there may be some measurement error in the categorization of phthalates exposure based on urinary metabolite concentrations because the correlations between measures of the same metabolite over relatively short intervals were moderate to low [9].

Given the observational nature of this study, we cannot conclude a causal relationship between late prenatal exposure to certain phthalates and reductions in IQ. Nevertheless, we have now observed consistent associations between exposure and outcomes measured at two time-points, one in the preschool years and one in the early school years, suggesting the results are not spurious and appear to be persistent. Indeed, the associations in the early school years are not diminished after control for MDI measured at age 3 years, suggesting a robust association. We note that the consistency of the associations over time has implications for public health and regulatory policy.

In conclusion, our analysis of the associations between prenatal phthalate exposure and IQ in the early school years showed significant decrements in IQ associated with two specific phthalates. These findings are important to inform policy makers of the potentially harmful effects of this class of chemicals.

## Supporting Information

Table S1 **Estimated coefficients of urinary phthalate concentrations in the linear model for WISC-IV when the children were 7 years of age.** ^a^Models include those with phthalate metabolite data at age 3. ^b^Models include those with phthalate metabolite data at age 5. *p<0.05, **p≤0.01. The model controlled for specific gravity (prenatal, age 3, and age 5 as appropriate), maternal IQ, ethnicity, alcohol use during pregnancy, education, marital status, total home score, and sex of child.
(DOCX)
