# Supplementary material for: Persistent Associations between Maternal Prenatal Exposure to Phthalates on Child IQ at Age 7 Years
Source: PLoS One. 2014 Dec 10;9(12):e114003. doi: 10.1371/journal.pone.0114003 (PMC4262205; doi:10.1371/journal.pone.0114003)
Supplement: Table S1 — Estimated coefficients of urinary phthalate concentrations in the linear model for WISC-IV when the children were 7 years of age. aModels include those with phthalate metabolite data at age 3. bModels include those with phthalate metabolite data at age 5. *p<0.05, **p≤0.01. The model controlled for specific gravity (prenatal, age 3, and age 5 as appropriate), maternal IQ, ethnicity, alcohol use during pregnancy, education, marital status, total home score, and sex of child. (DOCX) [file pone.0114003.s001.docx]

| **Supplemental Table. Estimated coefficients of urinary phthalate concentrations in the linear model for WISC-IV when the children were 7 years of age.** | | | | | | | | | | | | | | | | | |
| --- | --- | --- | --- | --- | --- | --- | --- | --- | --- | --- | --- | --- | --- | --- | --- | --- | --- |
|  | **N=241^a^** | | | **N=241** | | | | | **N=277**^b^ | | | **N=277** | | | | | |
|  | **Prenatal only** | | | **Prenatal and Age 3** | | | | | **Prenatal only** | | | **Prenatal and Age 5** | | | | | |
|  | **β** | **(95% CI)** | | **β** | | **(95% CI)** | **β** | **(95% CI)** | **β** | **(95% CI)** | | **β** | **(95% CI)** | | **β** | **(95% CI)** | |
|  | **Prenatal Coefficient** | | | **Prenatal Coefficient** | | | **Age 3 Coefficient** | | **Prenatal Coefficient** | | | **Prenatal Coefficient** | | | **Age 5 Coefficient** | | |
| **Metabolite** |  | |  |  |  | |  |  |  | |  |  | |  |  | |  |
| **CSFS** |  | |  |  |  | |  |  |  | |  |  | |  |  | |  |
| MnBP | -1.14 | | (-2.99, 0.72) | -1.10 | (-2.98, 0.78) | | -0.39 | (-2.14, 1.36) | -2.87 | | (-4.65,-1.08)** | -3.01 | | (-4.83,-1.19)** | 0.52 | | (-1.05, 2.09) |
| MBZP | -0.88 | | (-2.24, 0.47) | -0.43 | (-1.82, 0.96) | | -1.52 | (-2.71,-0.32)* | -1.33 | | (-2.61,-0.06)* | -1.32 | | (-2.64,-0.01)* | -0.19 | | (-1.40, 1.01) |
| MiBP | -1.42 | | (-3.26, 0.43) | -1.37 | (-3.25, 0.50) | | -0.37 | (-2.05, 1.31) | -2.47 | | (-4.07,-0.88)** | -2.65 | | (-4.29,-1.02)** | 0.62 | | (-0.92, 2.16) |
|  |  | |  |  |  | |  |  |  | |  |  | |  |  | |  |
| **CSPR** |  | |  |  |  | |  |  |  | |  |  | |  |  | |  |
| MnBP | -0.68 | | (-2.75, 1.39) | -0.50 | (-2.59, 1.60) | | -0.98 | (-2.93, 0.97) | -2.89 | | (-4.88,-0.90)** | -2.88 | | (-4.91,-0.85)** | -0.10 | | (-1.85, 1.66) |
| MBZP | -1.09 | | (-2.59, 0.42) | -0.56 | (-2.11, 0.98) | | -1.68 | (-3.01,-0.36)* | -1.48 | | (-2.89,-0.06)* | -1.44 | | (-2.90, 0.03) | -0.20 | | (-1.54, 1.13) |
| MiBP | -0.52 | | (-2.58, 1.54) | -0.33 | (-2.43, 1.76) | | -0.78 | (-2.66, 1.09) | -2.31 | | (-4.09,-0.53)* | -2.33 | | (-4.16,-0.51)* | 0.05 | | (-1.67, 1.77) |
|  |  | |  |  |  | |  |  |  | |  |  | |  |  | |  |
| **CSPS** |  | |  |  |  | |  |  |  | |  |  | |  |  | |  |
| MnBP | -1.38 | | (-3.59, 0.82) | -1.80 | (-4.00, 0.40) | | 1.66 | (-0.39, 3.70) | -2.29 | | (-4.44,-0.13)* | -2.13 | | (-4.33, 0.06) | -0.61 | | (-2.51, 1.28) |
| MBZP | -0.49 | | (-2.10, 1.13) | -0.67 | (-2.33, 0.99) | | 0.39 | (-1.04, 1.82) | -0.79 | | (-2.32, 0.74) | -0.51 | | (-2.08, 1.07) | -1.02 | | (-2.46, 0.42) |
| MiBP | -2.55 | | (-4.72,-0.37)* | -2.93 | (-5.11,-0.75)** | | 0.98 | (-0.97, 2.93) | -1.61 | | (-3.54, 0.31) | -1.40 | | (-3.37, 0.58) | -0.86 | | (-2.72, 1.00) |
|  |  | |  |  |  | |  |  |  | |  |  | |  |  | |  |
| **CSVC** |  | |  |  |  | |  |  |  | |  |  | |  |  | |  |
| MnBP | -0.85 | | (-2.64, 0.93) | -0.68 | (-2.49, 1.12) | | -0.71 | (-2.39, 0.96) | -1.64 | | (-3.28, 0.01) | -1.85 | | (-3.52,-0.18)* | 0.86 | | (-0.58, 2.30) |
| MBZP | -0.91 | | (-2.21, 0.40) | -0.62 | (-1.97, 0.72) | | -0.86 | (-2.01, 0.30) | -1.14 | | (-2.29, 0.02) | -1.27 | | (-2.46,-0.07)* | 0.36 | | (-0.73, 1.45) |
| MiBP | -1.16 | | (-2.93, 0.61) | -0.92 | (-2.72, 0.87) | | -1.02 | (-2.63, 0.58) | -2.19 | | (-3.64,-0.74)** | -2.46 | | (-3.94,-0.98)** | 1.01 | | (-0.38, 2.40) |
|  |  | |  |  |  | |  |  |  | |  |  | |  |  | |  |
| **CSWM** |  | |  |  |  | |  |  |  | |  |  | |  |  | |  |
| MnBP | -1.32 | | (-3.60, 0.96) | -1.27 | (-3.58, 1.03) | | -0.82 | (-2.97, 1.32) | -2.54 | | (-4.79,-0.29)* | -2.87 | | (-5.16,-0.59)* | 1.26 | | (-0.71, 3.23) |
| MBZP | -0.31 | | (-1.98, 1.35) | 0.41 | (-1.28, 2.10) | | -2.47 | (-3.92,1.01)** | -0.70 | | (-2.30, 0.90) | -0.75 | | (-2.40, 0.90) | -0.08 | | (-1.59, 1.43) |
| MiBP | -1.09 | | (-3.35, 1.18) | -1.14 | (-3.45, 1.16) | | -0.03 | (-2.09, 2.03) | -1.56 | | (-3.58, 0.45) | -2.01 | | (-4.07, 0.05) | 1.70 | | (-0.24, 3.63) |

^a^Models include those with phthalate metabolite data at age 3.

^b^Models include those with phthalate metabolite data at age 5.

**p* < 0.05, ***p* ≤ 0.01.

The model controlled for specific gravity (prenatal, age 3, and age 5 as appropriate), maternal IQ, ethnicity, alcohol use during pregnancy, education, marital status, total home score, and sex of child.
